# P-2073. Examining the Impact of Social Determinants of Health on Treatment Failure in US Female Patients Treated For Uncomplicated Urinary Tract Infection

**DOI:** 10.1093/ofid/ofaf695.2237

**Published:** 2026-01-11

**Authors:** Jacinda C Abdul-Mutakabbir, Seth Kuranz, Virginia Noxon-Wood, Karl M Kilgore, Meghan E Luck, Jeffrey J Ellis

**Affiliations:** Skaggs School of Pharmacy and Pharmaceutical Sciences, University of California, San Diego, La Jolla, CA, United States, San Diego, La Jolla, CA; Inovalon, Bowie, MD, United States, Bowie, Maryland; Inovalon, Bowie, MD, United States, Bowie, Maryland; Inovalon, Bowie, MD, United States, Bowie, Maryland; GSK, Brattleboro, VT; GSK, Brattleboro, VT

## Abstract

**Background:**

Evidence regarding the association of social determinants of health (SDOH) with treatment failure (TF) in uncomplicated urinary tract infection (uUTI) is lacking. This study assessed the impact of specific SDOH (including healthcare access and geographic location) on TF in patients (pts) with uUTI.

**Figure:**
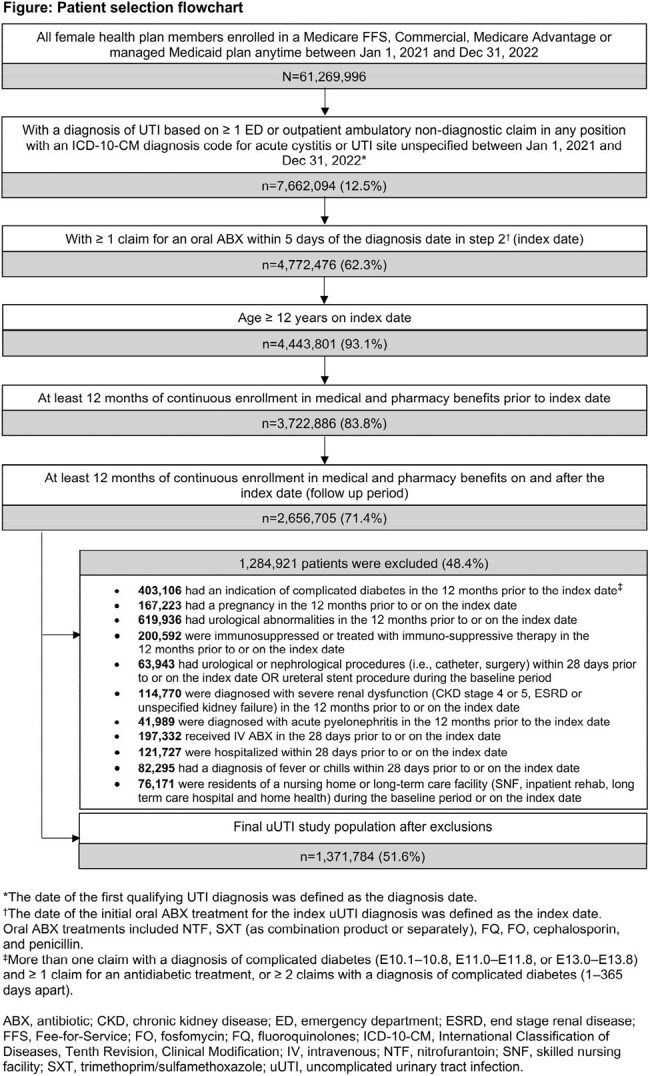

**Figure:**
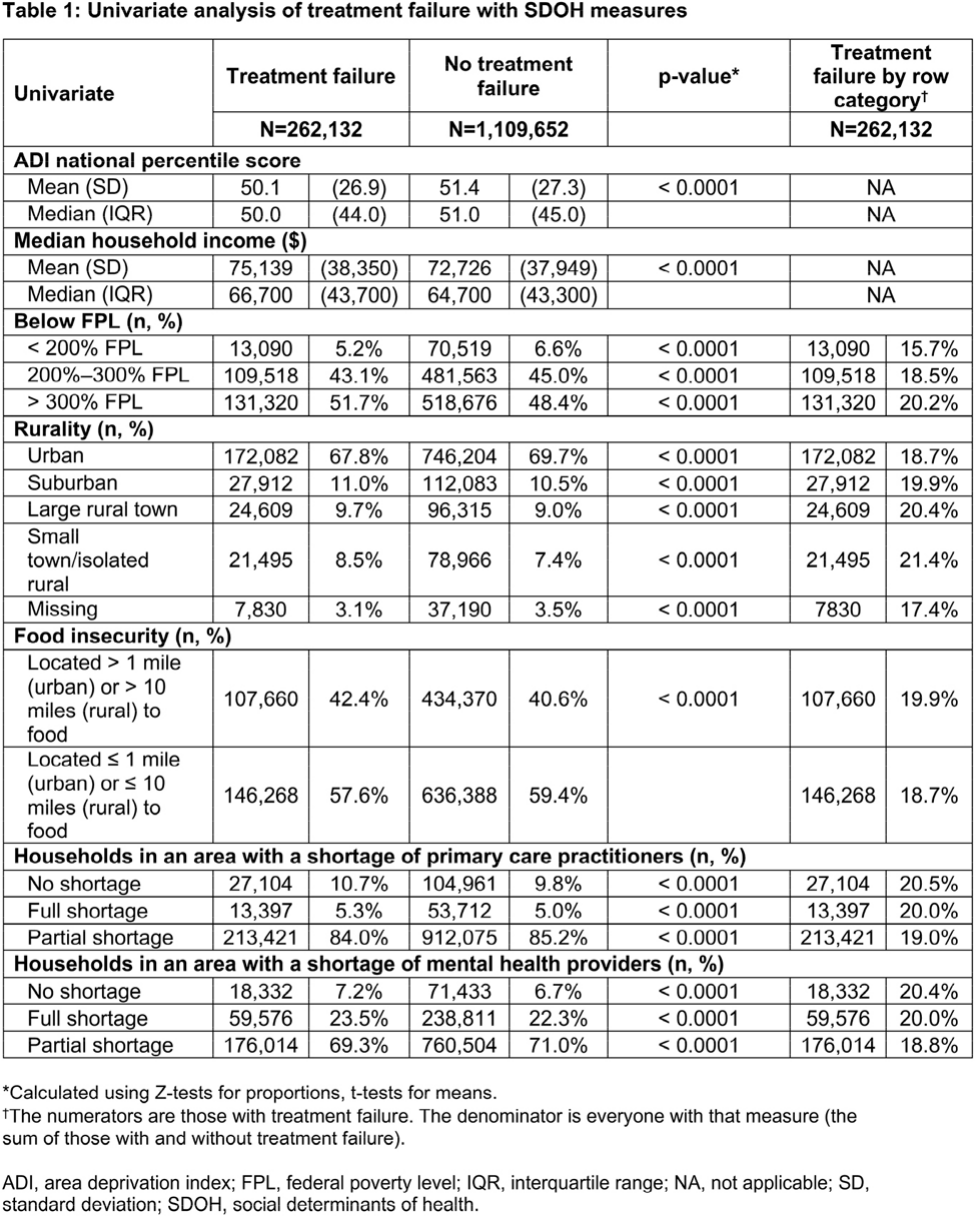

**Methods:**

Female pts aged ≥ 12 years with uUTI diagnosis (Dx) between Jan 2021 and Dec 2022, excluding complicated UTI, were assessed using Inovalon’s Medical Outcomes Research for Effectiveness and Economics (MORE^2^) Registry (commercial, Medicare Advantage, and managed Medicaid lives) and Centers for Medicare & Medicaid Services-sourced Medicare Fee-for-Service (FFS) Research Identifiable Files (all FFS beneficiaries, Figure). SDOH measures (Table 1) captured at the near neighborhood level (9-digit ZIP for MORE^2^ and 5-digit ZIP for Medicare FFS) were linked at the pt level to the Inovalon SDOH Data Warehouse. TF was defined as having a second oral antibiotic (ABX), intravenous ABX, or emergency department or inpatient stay with a primary Dx of UTI ≤ 28 days after the first oral ABX claim (index date; ± 5 days of uUTI Dx). Univariate comparisons between TF and no-TF cohorts were done using Z-tests for proportions and t-tests for means.

**Figure:**
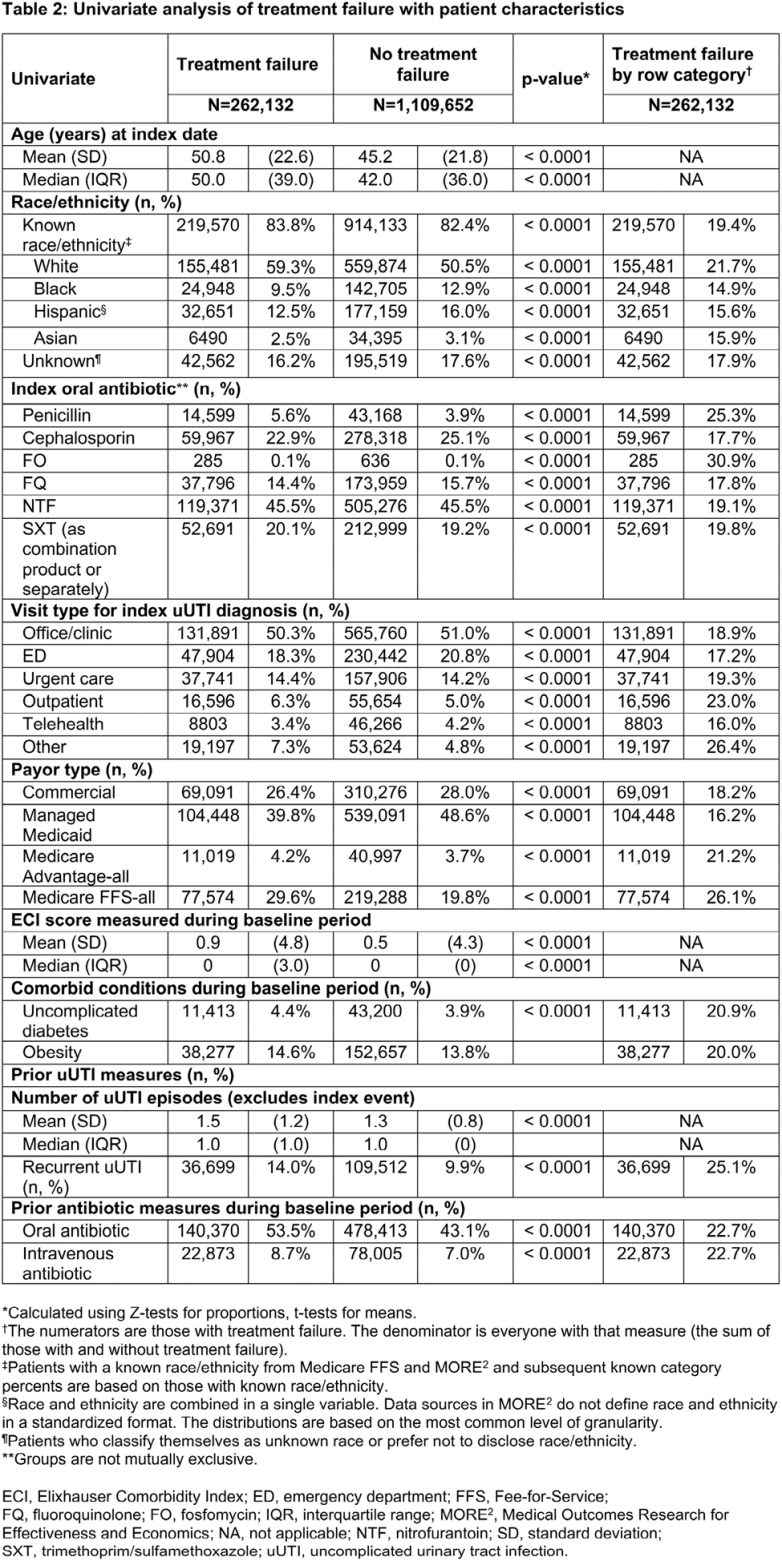

**Figure:**
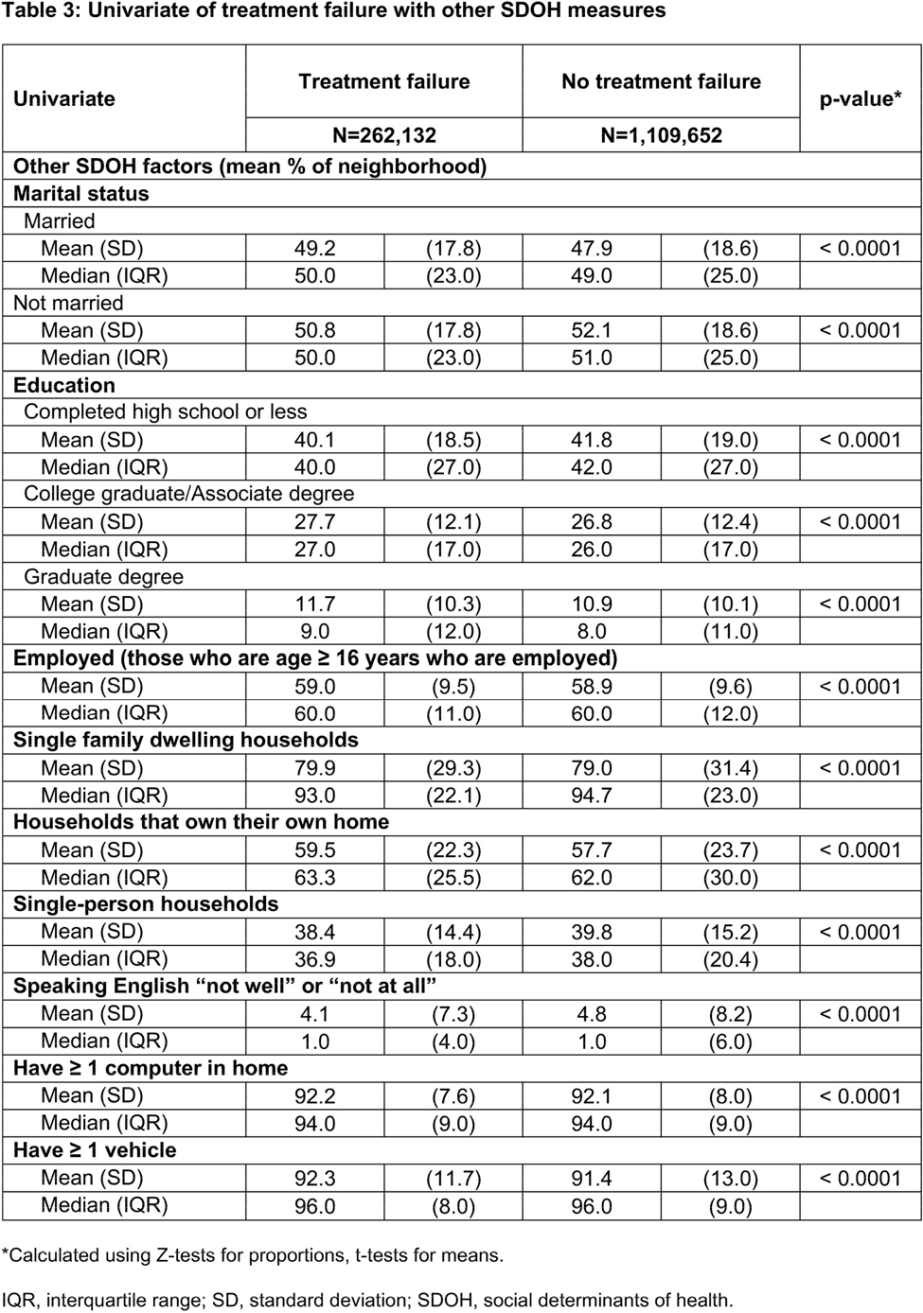

**Results:**

Overall, 1,371,784 female pts with uUTI were identified (19.1%, n=262,132 in TF cohort). The TF cohort was older than the no-TF cohort (mean [median] age, 50.8 [50.0] vs 45.2 [42.0]) and had a higher percentage of Medicare FFS versus no-TF (29.6% vs 19.8%). Small absolute differences were observed between TF and no-TF cohorts across SDOH measures (Table 1) and pt characteristics (Table 2). Versus the no-TF cohort, the TF cohort exhibited higher levels of residence in rural settings and areas with full shortages in primary and mental health providers. The TF cohort also faced higher levels of food insecurity and chronic disease burden, including diabetes and obesity, as well as other SDOH factors (Table 3).

**Conclusion:**

Subtle differences in SDOH between female pts with TF and no-TF for uUTI were found. Further investigation is needed to determine interactions between pt characteristics, clinical factors, and other SDOH and the impact of those interactions on TF.

Funding: GSK study 222864.

**Disclosures:**

Jacinda C. Abdul-Mutakabbir, PharmD, MPH, CSL Seqirus: Advisor/Consultant|CSL Seqirus: Honoraria|GSK: Advisor/Consultant|Shionogi: Advisor/Consultant|Shionogi: Honoraria Seth Kuranz, PhD, Inovalon: Employee of Inovalon, a consulting company that received funding from GSK to conduct this study. Virginia Noxon-Wood, PhD, Inovalon: Employee of Inovalon, a consulting company that received funding from GSK to conduct this study. Karl M. Kilgore, PhD, Karl M. Kilgore: Employee of Inovalon, a consulting company that received funding from GSK to conduct this study. Meghan E. Luck, PharmD, GSK: Employee|GSK: Stocks/Bonds (Public Company) Jeffrey J. Ellis, PharmD, MS, GSK: Employee|GSK: Stocks/Bonds (Public Company)

